# Insulin in the Brain: Its Pathophysiological Implications for States Related with Central Insulin Resistance, Type 2 Diabetes and Alzheimer’s Disease

**DOI:** 10.3389/fendo.2014.00161

**Published:** 2014-10-09

**Authors:** Enrique Blázquez, Esther Velázquez, Verónica Hurtado-Carneiro, Juan Miguel Ruiz-Albusac

**Affiliations:** ^1^Departamento de Bioquímica y Biología Molecular III, Facultad de Medicina, Universidad Complutense, Madrid, Spain; ^2^The Center for Biomedical Research in Diabetes and Associated Metabolic Disorders (CIBERDEM), Madrid, Spain; ^3^Instituto de Investigación Sanitaria del Hospital Clínico San Carlos (IdiSSC), Madrid, Spain

**Keywords:** brain, insulin, receptors, biological actions, pathophysiological implications, central insulin resistance, type 2 diabetes, Alzheimer’s disease

## Abstract

Although the brain has been considered an insulin-insensitive organ, recent reports on the location of insulin and its receptors in the brain have introduced new ways of considering this hormone responsible for several functions. The origin of insulin in the brain has been explained from peripheral or central sources, or both. Regardless of whether insulin is of peripheral origin or produced in the brain, this hormone may act through its own receptors present in the brain. The molecular events through which insulin functions in the brain are the same as those operating in the periphery. However, certain insulin actions are different in the central nervous system, such as hormone-induced glucose uptake due to a low insulin-sensitive GLUT-4 activity, and because of the predominant presence of GLUT-1 and GLUT-3. In addition, insulin in the brain contributes to the control of nutrient homeostasis, reproduction, cognition, and memory, as well as to neurotrophic, neuromodulatory, and neuroprotective effects. Alterations of these functional activities may contribute to the manifestation of several clinical entities, such as central insulin resistance, type 2 diabetes mellitus (T2DM), and Alzheimer’s disease (AD). A close association between T2DM and AD has been reported, to the extent that AD is twice more frequent in diabetic patients, and some authors have proposed the name “type 3 diabetes” for this association. There are links between AD and T2DM through mitochondrial alterations and oxidative stress, altered energy and glucose metabolism, cholesterol modifications, dysfunctional protein O-GlcNAcylation, formation of amyloid plaques, altered Aβ metabolism, and tau hyperphosphorylation. Advances in the knowledge of preclinical AD and T2DM may be a major stimulus for the development of treatment for preventing the pathogenic events of these disorders, mainly those focused on reducing brain insulin resistance, which is seems to be a common ground for both pathological entities.

## Introduction

Although the control of peripheral glucose homeostasis is one of the main functions of insulin, its action on the brain is now also being studied carefully, as it is considered an insulin-sensitive organ because insulin receptors (IR) and their signal transduction pathways have been identified in several regions of the brain that mediate important physiological effects on this organ, such as neuronal development, glucoregulation, feeding behavior, and body weight, as well as cognitive processes, including attention, executive functioning, learning, and memory (1).

## Presence of Insulin in the Brain: Is Insulin Synthesized in the Brain?

In the late 1970s, the central nervous system (CNS) was not considered to be an insulin-dependent tissue, but it is now well known that insulin plays a major physiologic role in this tissue and its disturbances, being involved in certain neurodegenerative states, such as Alzheimer’s disease (AD). The presence of insulin in the brain was first detected by Havrankova et al. (2), who used radioimmunoassay to determine high levels of insulin in brain extracts. Likewise, they reported that insulin content in the brain was independent of the peripheral insulin, since circulating insulin levels had no effect on the brain’s insulin concentration (3). In addition, high insulin concentrations had also been reported not only in the human brain but also in several experimental animals (4).

The detection of insulin in cerebrospinal fluid (CSF) should not be interpreted as a robust indication of blood–brain barrier (BBB) transport. There is a blood:CSF barrier located at the choroid plexus, and insulin entering the brain from the blood cannot be expected rapidly be excreted in CSF (5, 6).

The presence of high concentrations of insulin in brain samples has raised the question of its origin. This question continues to be one of the most debated aspects of the research into cerebral insulin. Previous findings support the hypothesis that, at least in part, “brain insulin” is produced in the CNS. However, as posited by Havrankova et al. (2), other sources for cerebral insulin should be considered, such as its peripheral origin and then crossing the BBB, versus a central origin, or both.

### Peripheral origin of insulin

The notion that insulin could cross the BBB was first suggested by Margolis and Altszuler (7), who showed that insulin levels in the CSF of rats increased slightly after peripheral infusions of this hormone, suggesting that insulin crossed the BBB possibly by means of a saturable transport system. These results were later confirmed in dogs after the iv administration of insulin (8). They found a large, rapid increase in blood insulin, but a relatively small increase in the hormone in the CSF. These findings confirmed a non-linear correlation between plasma and CSF levels of insulin, providing the first evidence for a saturable transport system for insulin from blood to the brain. Although there is no direct evidence on whether the insulin transport system and the IR are the same protein, this seems to be widely assumed (9), as they have similar physicochemical properties (saturability, specificity, affinity, immunoneutralization, cooperative interactions, and kinetics of dissociation) to IRs (10) in typical target tissues (11). On the other hand, differences in the activity of the BBB transporter system may be responding to regional differences in insulin permeability, and also to the hormone concentration, recording the highest values in the pons, medulla, and hypothalamus, and the lowest in the occipital cortex and thalamus (12). This insulin transport may be regulated by multiple factors, such as glucocorticoids (13), or in several pathophysiological situations, such as fasting and re-feeding (14), obesity (15), and hibernation (16), as well as during aging and in patients with diabetes mellitus (DM), and AD (17, 18).

### Central origin of insulin

The local production of insulin in the CNS has also been widely studied, and suggestions of possible insulin biosynthesis in the brain have been based on different experimental evidence, as cited below.

#### Detection of C-peptide in the brain

Immunoreactive insulin and C-peptide were found in the brain from human cadavers (19), recording concentrations that were much higher in the brain than in the blood, with the highest content in the hypothalamus. In addition, C-peptide concentration decreased after 72 h of fasting, and increased after oral glucose administration in both plasma and hypothalamus (20). On the other hand, C-peptide was found in the post-mortem brain cortex of elderly and AD subjects, showing for the first time a direct correlation between C-peptide concentrations and a decrease in the number of brain IRs (21). These findings led the authors to conclude that, at least in part, cerebral insulin was a product of the brain itself.

#### Detection of insulin mRNA in certain brain regions

Evidence of the presence of insulin mRNA was found in the periventricular nucleus of the rat hypothalamus by *in situ* hybridization (22). Furthermore, the use of RNase-protection and sensitive reverse transcription-polymerase chain reaction (RT-PCR) assays led to the detection of insulin II mRNA in the brains of fetal, neonatal, and adult rats, which suggests that the ancestral insulin II gene expression in rat brains belongs to the pre-pancreatic stage of embryonic development (23). Likewise, insulin mRNA was located in the CA1 and CA3 regions of the hippocampus, in the dentate gyrus, and in the granule cell layer of the olfactory bulbs of the neonatal rabbit brain by *in situ* hybridization experiments (24).

#### Experimental approaches with brain cell cultures

Evidence supporting the synthesis of insulin in the CNS has also been obtained from brain cell cultures. Thus, incubation with [^3^H]valine resulted in the incorporation of radioactivity into immunoprecipitable insulin, and with cycloheximide caused an 80% decrease in the number of insulin-like immunoreactive neurons from primary cultures of rat brain (25). In addition, two main forms of the immunoreactive insulin (IRI) were detected in cultures from fetal mouse brain. The major component resembling pro-insulin was converted by trypsin into the minor form, which was similar to real pancreatic insulin (26). Likewise, using immunohistochemical and *in situ* hybridization techniques it has been showed the ability of fetal neuron cell cultures to produce and secrete an insulin-like mRNA and an insulin-like substance (ILS) that was indistinguishable from real insulin (27). On the other hand, the novo synthesis and secretion of insulin by central mammalian neurons in both neuron-enriched and glial-enriched postnatal rabbit brain cell cultures was studied (24), indicating that specific neurons, but not astrocytes, produced an extracellular secretion of immunoprecipitable insulin.

The molecular mechanisms involved in the production and secretion of insulin in the CNS reveal similarities between beta cells and neurons, particularly in relation to ATP-sensitive K^+^ channel depolarization (28). Both beta cells and neurons are electrically excitable and respond to hormonal stimuli and glucose by depolarization and exocytosis. The depolarization by potassium ions (in the presence of calcium) of primary cultures of neuronal cells caused a threefold stimulation of insulin release. This depolarization-induced release of insulin was inhibited by cycloheximide, and was specific for neurons, but not for astrocytes (29). Similarly, insulin was also released from adult rat brain synaptosomes under depolarizing conditions, and depending on calcium influx, which suggested that insulin was stored in the adult rat brain in synaptic vesicles within nerve endings, from which it can be mobilized by exocytosis related to neural activity (30). In synaptosomes, it has been shown that insulin secretion was increased by glucose, and that the addition of the glycolytic inhibitor, iodoacetic acid (IAA), produced a 50% decrease in the glucose-induced release of IRI, suggesting that, as occurs in the pancreas, glucose metabolism is also involved in brain insulin release (31). These results imply that the brain itself might synthesize some portion of the insulin detected locally, which is not an unusual occurrence (32).

### Effect of insulin on brain endothelial cells and blood–brain barrier cell function

**Figure 1:**
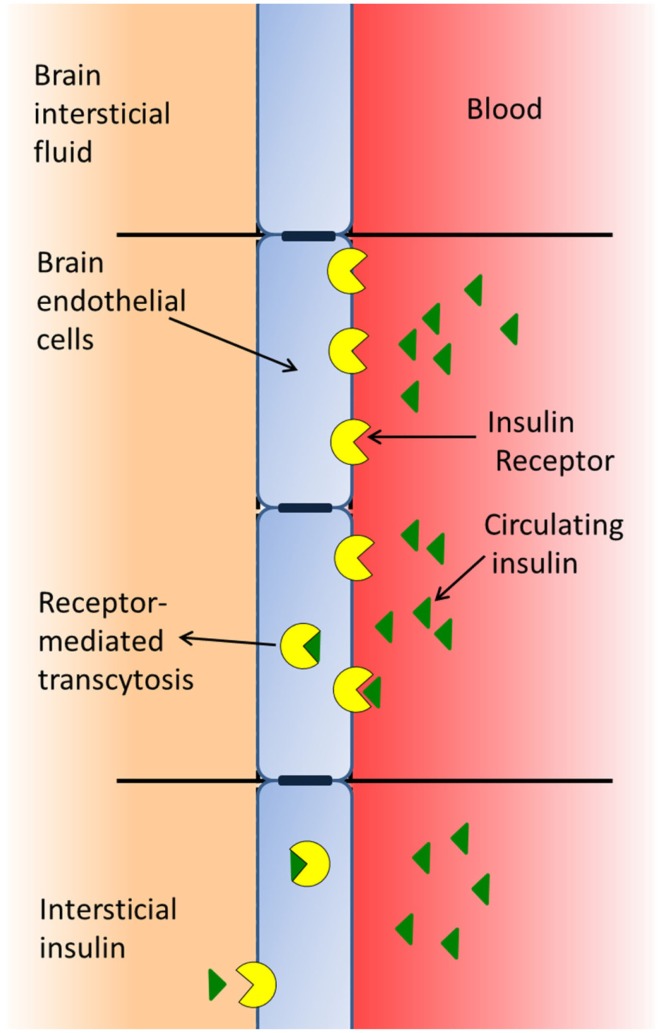
**Insulin of peripheral origin may pass through the blood–brain barrier using a receptor-mediated transport system**.

The BBB is formed by a type of brain endothelial cell (33) that is unique, since the cell membranes are exposed both to the blood stream and to the CNS, whereby these cells receive signals from both the periphery and the CNS (17). BECs have insulin-binding sites that appear to have two distinct functions: as transporters of insulin across the BBB (Figure 1) and as classic receptors (34), both affecting the function of the barrier cell by activating intracellular machinery and mediating the effects of insulin on these cells, such as the increase in the transport of tyrosine and tryptophan (35), azidothymidine (36), and leptin (37) from blood to brain. In addition, insulin modifies the expression and/or activity of certain efflux transporters. Thus, insulin induces P-glycoprotein expression (the 170-kDa protein product of the multidrug resistance one gene), which plays an important role in the integrity of the BBB, protects the brain from many exogenous toxins (38), and suppresses the expression and function of the breast cancer resistance protein (39). Likewise, insulin induces neurochemical modifications in brain microvessels by inhibiting the activity of alkaline phosphatase (40), and increasing the expression and activity of the glutamate–cysteine ligase catalytic subunit by activating the antioxidant response element-4 (41). In addition, insulin inhibits the activity of the serotonin receptor 5-HT_2c_ in choroid plexus, showing that this G-protein-coupled receptor (GPCR) is modulated by the tyrosine-kinase receptor-MAP kinase pathway (42).

On the other hand, the insulin-degrading enzyme (IDE) is present in several brain regions, including multiple cortical areas, hippocampus, cerebellum, and brain stem. At cellular level, IDE was confined mainly to neurons, but it was also present in oligodendrocytes, choroid plexus, and some blood vessel endothelial cells (43). IDE is upregulated by exposure to low levels of amyloid-beta peptide (Abeta), which may be an important therapeutic target because of its role in the degradation of Abeta and other substances (44).

## Mechanisms of Insulin Signal Transduction in the Brain

### Brain insulin receptors

The single gene of the human IR, located on chromosome 19p13.2–19p13.3, has 22 exons (11 each for coding the α and β subunits). Two isoforms of the precursor protein are generated by the alternative splicing of +/− exon 11 (IR-B/IR-A, respectively) in a tissue-specific manner. This exon encodes a small amino acid sequence that is located at the C-terminal of the extracellular α-subunit (45). In humans, IR-B (the longer isoform) is the most prominent isoform in classical insulin-sensitive tissues, skeletal muscle, adipose tissue, and liver, as opposed to IR-A in the brain (46–48).

The heterotetrameric IR is composed of two ligand-binding sites, disulfide-linked extracellular α subunits, which are linked by disulfide bonds to two membrane-spanning β subunits. The α subunit is predominantly hydrophilic in nature, lacks membrane anchor regions, and contains 15 potential N-glycosylation sites and 37 cysteine residues. The β subunit contains a portion that is extracellular, a portion that comprises the transmembrane region of the receptor, and a portion that is intracellular, and which possess inherent tyrosine-protein-kinase activity (49).

Although the presence of IRs in many tissues in the periphery, and their main function of mediating glucose transport into cells, was well known, the existence of IRs within the brain was poorly understood, and their function sometimes seemed to be something of an enigma because brain cells are not fully reliant upon insulin for glucose supply inasmuch as they have insulin-independent means of obtaining glucose (49). However, we now know that insulin signaling in the brain affects several important functions.

Studies on the presence of IRs in the CNS began in the early 1970s with the observation that systemic glucose concentration decreased after the injection of 500 μU of insulin into the carotid artery of rats (50), and through the report of specific binding of radiolabeled ^125^I-insulin in a crude membrane preparation of several tissues from monkeys, rats, and pigeons (51). The hepatic carbohydrate metabolism was thus reported to be under cholinergic influence through efferent neural pathways, and not due to a modification of pancreatic hormone secretion. IR was located and quantified in the CNS for the first time in 1978 (52), being present in membrane preparations from the brain at all stages of the development studied (53). Since then, a wide but uneven distribution of IR in the CNS has been reported. Accordingly, it was shown that membrane preparations from the hypothalami specifically bound greater [^125^I]insulin than membranes from the cortex and thalamus, and that this binding was higher for preparations from the anterior rather than the posterior portions of the hypothalamus (54). Likewise, the binding of [^125^I]insulin was high not only in all olfactory areas and in closely related limbic regions, but also in the neocortex and accessory motor areas of the basal ganglia, hippocampus, cerebellum, and choroid plexus, which suggested a neuromodulatory function for insulin in the brain (55). When IRs were quantified by autoradiography and computerized densitometry, the highest concentrations were detected in regions concerned with olfaction, appetite, and autonomic functions, all of which contain dendritic fields receiving rich synaptic input (56). *In situ*, hybridization showed that IR mRNA was the most abundant in the granule cell layers of the olfactory bulb, cerebellum, dentate gyrus, in the pyramidal cell body layers of the piriform cortex, hippocampus, in the choroid plexus, and in the arcuate nucleus of the hypothalamus; these findings were consistent with the distribution of IR binding (57). Interestingly, the expression of IR mRNA seems to be higher in the brain from obese (fa/fa) Zucker rats as compared with lean (Fa/−) age-matched controls (58). However, brain homogenates from normal and streptozotocin-induced diabetic rats showed similar specific insulin-binding, which indicated the absence of the upregulation of these receptors (59).

As compared with IRs, IGF1 receptors (IGF1R) are also widespread throughout the rat brain, but they have a distinct distribution, with a high concentration in regions concerned with olfaction, autonomy, and sensory processing, as well as in the pituitary gland, where they are involved in the regulation of growth hormone release (60). What’s more, the existence has been reported of a differential expression of both IGF-1R and IR in the left–right of male–female developing rat hippocampus, which may be responsible for the etiology of several mental health disorders, as well as sex differences in hippocampal-associated behaviors such as spatial learning strategies and stress response (61).

Insulin receptors are also widely distributed in the human brain, with the highest specific binding of [^125^I]labeled human insulin in homogenates prepared from hypothalamus, cerebral cortex, and cerebellum obtained post-mortem from non-diabetic subjects (62). Iodinated insulin-binding to synaptosomal membranes within the human cortex was found to be a function of age. Binding to IR was observed as early as week 14 of gestation, with a slight decrease around week 30, and a marked decrease after birth (63).

Brain IRs have similar kinetics and pharmacological properties to those described in peripheral tissues (64), although they differ in molecular size (as indicated, the α subunits of brain IR, named IR-A, are smaller than the α subunits of peripheral ones, called IR-B), degree of glycosylation (being higher in peripheral than in brain IR), and antigenicity. In addition, regulation by insulin also occurs in a different way, thus, while peripheral IRs are downregulated in response to insulin excess, their counterparts in the brain do not record such downregulation (65). Receptor heterogeneity is a powerful principle that allows the independent and specific regulation of cellular functions via identical hormones or second messengers. Furthermore, the presence of different receptor isoforms allows an independent regulation of their expression by different mechanisms (66). Some regions show a marked difference in IR density between the embryonic and adult brain, which may play a developmental role. Thus, high concentrations of IR are found in the thalamus, caudate–putamen, and some mesencephalic and brainstem nuclei during neurogenesis, but these same areas have a low IR density in adult rat brains (67).

### Brain insulin receptor signaling

**Figure 2:**
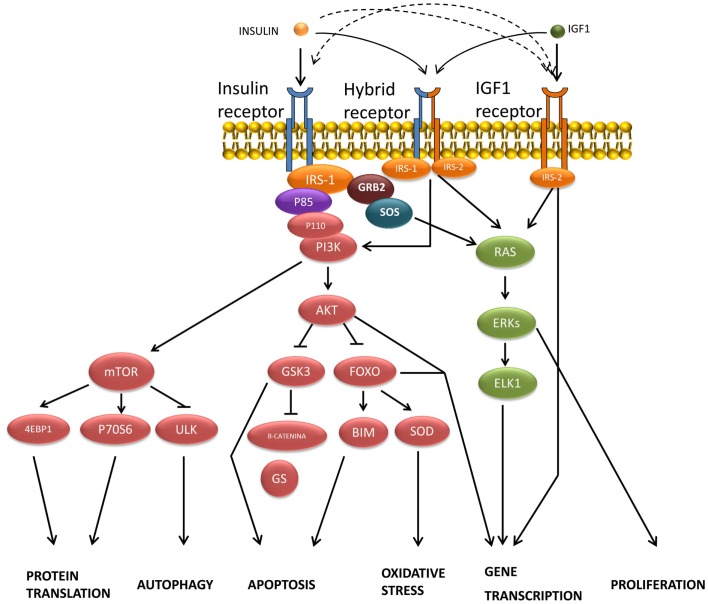
**Transduction of signals and biological actions induced by insulin or IGF-1 through their receptor or through their hybrid receptors**.

Insulin-binding to α subunits of the IRs triggers the activation of the β subunit tyrosine-kinase activity by stimulating the phosphorylation of its own receptor in both neuronal and glial cells (68). In most higher animals, the mechanism of insulin signal transduction (Figure 2) is modulated through the tyrosine phosphorylation of cellular substrates, including several insulin receptor substrates (IRSs) (69), as well as other scaffold proteins (70), which initiate divergent signal transduction pathways (71). Likewise, following the binding of insulin, aggregated IRs are rapidly internalized into the cell by a process that at least in part involves coated pits and vesicles (72). It has been suggested that aggregation or internalization could be essential for insulin signaling (73). The internalized IRs can then be degraded or recycled back to the cell membrane.

Most insulin responses are mediated by IRS-1 and IRS-2. IRS-1 controls body growth and peripheral insulin action, while IRS-2 regulates brain growth, body weight control, glucose homeostasis, and female fertility (74). IRS proteins are composed of an NH_2_-terminal pleckstrin homology (PH) domain adjacent to a phosphotyrosine-binding (PTB) domain, and followed by a tail containing numerous tyrosine and Ser/Thr phosphorylation sites (75). The Tyr phosphorylation sites coordinate downstream signaling cascades by binding the SH2 domains present in common effector proteins, including enzymes (the phosphoinositide 3-kinase, PI3K; the phosphatase SHP2; or the tyrosine-kinase Fyn) or adapters (SOCS1, SOCS-3, GRB2, and others) (70, 74). By contrast, the specific serine phosphorylation of the IRS-1/2 by the c-Jun N-terminal kinase (JNK1) and other protein kinases inhibits insulin-stimulated tyrosine phosphorylation, which correlates closely with insulin resistance (76). Likewise, the ubiquitin-mediated degradation of IRS-1/2 also promotes insulin resistance (77). However, the agonists that increase IRS-2 expression through cAMP production and CREB activation improve insulin signaling (78).

The synapse is the primary locus of cell–cell communication in the nervous system. It has been reported that IR was co-expressed with the insulin receptor tyrosine-kinase substrate p58/53 (IRSp53) in the synapse-rich molecular layer and in the granule cell layer of the cerebellum, as well as in the synapses of the cultured hippocampal neuron, which suggested that these molecules could be part of an insulin-dependent signaling pathway at the post-synaptic apparatus (79). IRSp53, which is phosphorylated upon stimulation with insulin (80, 81), is a key factor in cytoskeleton reorganization that mediates neurite outgrowth (82), being also involved in several neurodegenerative disorders (83), because IRSp53-deficient animals record cognitive deficits in the contextual fear-conditioning paradigm (84).

The association of IRS proteins and PI3K triggers the activation of this enzyme, which phosphorylates an inositol phospholipid in the plasma membrane, named PI (4,5)P_2_, to PI (3,4,5)P_3_, which recruits both the Ser/Thr kinase PDK (3-phosphatidylinositol-dependent protein kinase) and protein kinase B (PKB or Akt) to the plasma membrane, where Akt is activated by PDK1- and PDK2-mediated phosphorylation (85). This signaling pathway is antagonized by the action of the phospholipid phosphatases PTEN or SHIP2. Akt phosphorylates several substrates, including TSC2 (tuberous sclerosis complex, tuberin), which finally activates the mammalian target of rapamycin (mTOR) and provides a direct link between insulin signaling and nutrient sensing (70, 86). In addition to the IRS/PI3K/Akt, a second signaling pathway has been reported in peripheral tissues for the translocation of the glucose transporter GLUT-4 by insulin, involving other substrates of IR such as Cbl and APS. Following the recruitment of several proteins, including TC10, into the lipid raft, the trafficking of GLUT-4 vesicles is stimulated until their fusion with the plasma membrane (71, 85).

Mitogen-activated protein kinase is another signaling pathway activated by insulin through tyrosine phosphorylation of certain prototypical signaling adaptors such as Gab-1/Shp2, Shc/Grb2, and SOS/Grb2, which activate the small G-protein Ras by stimulating GDP:GTP exchange. Raf activation then takes place through a multi-step process (87), initiating an activation cascade of several protein kinases that include MAPK/ERK kinase (MEK) and extracellular signal-regulated kinase (88). ERK phosphorylates and activates several cytosolic proteins including p90^rsk^ (89) cytoskeletal proteins, phospholipase A2 (PLA2), and signaling proteins, such as tyrosine-kinase receptors, estrogen receptors, SOS, and STATs (signal transducer and activator of transcription proteins). ERK also enters the nucleus, where it controls gene expression by phosphorylating transcription factors such as Elk-1 and other Ets-family proteins (18, 70).

Some brain dysfunction might result not only from an aberrant IR expression or function that occurs either during development or later, but also from single-point mutations, such as F382V (delayed transport of IR components to cell surface); R735S (insulin resistance due to the inhibition of precursor processing); L1018A (absence of tyrosine-kinase activity); and Y960F (multiple functional defects) (49).

## Insulin Actions in the Brain

### Effects on energy expenditure, glucose homeostasis, and feeding behavior

Although the brain uses ketone bodies during starvation, glucose is its main fuel, which is needed in a continuous and permanent supply (90). Besides being an energy substrate, glucose is a signaling molecule involved in glucoregulatory mechanisms of primary functional concern to provide an uninterrupted glucose supply to the CNS and meet the metabolic needs of peripheral tissues. Given the vital importance of the continuous supply of glucose to the brain and the high prevalence of DM, the possible lack of insulin-dependent glucose uptake may be considered as an advantage.

The brain has two groups of glucose-sensitive neurons named glucose-excited (GE) and glucose-inhibited (GI) by rises and falls in glucose concentrations, respectively. These neurons are involved in the control of feeding, energy expenditure, and glucose homeostasis (49) and in addition the glucokinase acts as a glucose sensor in those neurons, facilitating the control of food intake (91–94). These various glucoregulatory functions are usually secondary to glucose uptake, a step that in most tissues is controlled by the level of glucose transporter (Table 1) and glucose sensor expression (95). GLUT-1, the more abundant glucose transporter in the brain, is expressed as two isoforms that differ in their degree of glycosylation. The 45-kD isoform expressed in astrocytes is resistant to both hypoglycemia and hyperglycemia, while the expression of the 55-kD isoform, originally located in the capillary endothelial cells, is increased under conditions of hypoglycemia, but remains unchanged during hyperglycemia. GLUT-1 has a widespread distribution in the brain (96), where it seems to have tissue-specialized functions, and some isoforms could be sensitive to acute insulin regulation (49). GLUT-2 is expressed in several neuronal populations, including specific neurons in the hypothalamus such as the paraventricular nucleus, the arcuate nucleus, and the lateral region (97, 98), where GLUT-2 is co-expressed with glucokinase (49, 93) and sulfonylurea receptor-1 (SUR1) (99). GLUT-3, the major glucose transporter in the neurons of the cerebellum, striatum, cortex, and hippocampus (100), has also been detected in brain glial and endothelial cells (101) operating at lower glucose levels, which is important given that the glucose concentration in the brain interstitium is relatively low as compared to in the blood.

**Table 1:** **Main glucose transport (GLUT) isoforms in the brain**.

Glucose transport isoforms	Location	Cell types	Abundance	Control
GLUT-1	Ubiquitous	Glia and endothelial	Very abundant	Hypoglycemia, insulin
GLUT-2	Hypothalamus	Neurons, glia, and tanycytes	Limited	
GLUT-3	Cerebellum, striatum, cortex, and hippocampus	Neurons, glia, and endothelial	Very abundant	
GLUT-4	Olfactory bulb, hippocampus (dentate gyrus), and hypothalamus cerebellum	Neurons and glia	Selective areas	Glucose, insulin and exercise training
GLUT-8	Hypothalamus, cerebellum, brainstem, hippocampus, dentate gyrus, amygdala, and primary olfactory cortex	Neurons: bodies and proximal apical dendrites	Limited	Glucose

In contrast with peripheral tissues, the brain is considered an insulin-insensitive organ because GLUT-4 is present at low level and it does not seem to be significantly regulated by insulin. Thus, GLUT-4 was located in selective areas of the brain, including the olfactory bulb, dentate gyrus of the hippocampus, hypothalamus, and cortex, but at low amounts compared to the other isoforms, GLUT-1 and GLUT-3. As in those tissues, GLUT-4 was also located in both the plasma membrane and cytoplasm, which could suggest that a readily mobilizable pool was available for translocation to the plasma membrane (102). Surprisingly, in cerebellar membranes, GLUT-4 was present in significant amounts and its expression was insulin-dependent (103). In addition, the trafficking of GLUT-4 to the plasma membrane was modulated in the cerebellum, cortex, and hippocampus under conditions that increased plasma insulin levels (104), such as after peripheral glucose administration. Also, as GLUT-4, GK, and IR were co-expressed in both GE and GI hypothalamic neurons, these findings could suggest that this brain region, may experience stimulation of glucose uptake in response to insulin (105). However, the observation that GE and GI neurons respond to alterations of ambient glucose levels in the complete absence of insulin (97, 98, 106), and that insulin fails to induce neuronal glucose uptake in hippocampal formation, and that IR activation with insulin in humans has no effect on AS160-dependent GLUT-4 translocation (104), it seems possible to conclude that insulin-mediated glucose transport is at least not required by glucosensing neurons.

The neuron-specific glucose transporter GLUT-8, which has limited association with the plasma membrane in the CNS under physiological settings or in experimental models of type 1 diabetes (107), is expressed in bodies and in the most proximal apical dendrites of several brain areas (108), including both excitatory and inhibitory neurons in the hippocampus (109). Its functional role is still little known, but being an insulin-responsive isoform, it may play a role in augmenting substrate delivery under conditions of increased demand (110). As GLUT-8 is present in the rough endoplasmic reticulum and cytosol, a new role for this glucose transport has been proposed. Thus, since glucose is released from oligosaccharides during protein glycosylation events that occur in the rough endoplasmic reticulum, GLUT-8 may transport glucose out of the rough endoplasmic reticulum and into the cytosol, and thereby contribute to glucose homeostasis in hippocampal neurons, which is impaired under hyperglycemic/insulinopenic conditions (111, 112). As with GLUT-4, glucose administration also stimulates GLUT-8 trafficking from the cytosol to the rough endoplasmic reticulum, but does not result in GLUT-8 association with the plasma membrane.

Although insulin does not induce a significant glucose uptake in the brain as compared with peripheral tissues, it may play other important roles in glucose homeostasis. Thus, the experimental inhibition of insulin action in the hypothalamus, or the direct stimulus of the arcuate nucleus with this hormone, induces a reduction in insulin’s ability to block the production of liver glucose (113). To produce this effect, insulin acts through its own receptors in the liver and hypothalamus. Accordingly, a reduction in insulin sensitivity in the hypothalamus might lead to the diminished efficiency of this hormone in blocking glucose formation (114), which might contribute to the hyperglycemia of diabetic patients (115, 116). The effect of insulin on hypothalamic glucose-sensitive neurons might be to induce an opening of the ATP-sensitive K^+^-channels (117), causing a cell-hyperpolarization that ameliorates the functional capacity to modify the glucose response of these glucose-sensitive cells (118). The signals generated in this process are transmitted to the motor nucleus of the vagus nerve that carries this information to the liver, which produces the appropriate response. In fact, the resection of the liver branch of the vagus nerve induces a decrease in the insulin inhibitory effect on glucose hepatic production (116). All these findings lend support to the report by Claude Bernard, who in 1855 (119) showed that puncturing the fourth cerebral ventricle produced glycosuria in mice, which gives rise to the assumption that the brain is involved in glucose homeostasis.

We now consider the existence of specific populations of neurons involved in energy homeostasis, located on the so-called satiety and hunger networks rather than centers. They contain orexigenic and anorexigenic molecules, and their receptors are closely interrelated with metabolic sensors such as the glucose transporter isoform GLUT-2, glucokinase (92–94), AMPK, PASK, and others. They generate integrated responses to afferent stimuli related to modifications in metabolites or in fuel storage. It is accepted that the cells of several hypothalamic nuclei detect circulating satiety signals and transmit this information to other brain areas. The interactions among orexigenic and anorexigenic molecules and transmitters located in different hypothalamic nuclei may induce a characteristic feeding behavior. The activation of the PI3K pathway is common to insulin, leptin, and serotonin, and it is believed that the dialog between these biomolecules is potentially a major event in the pathophysiology of the brain from a general perspective, and in the control of food intake in particular. Insulin is a powerful anabolic hormone in the periphery, and behaves as a catabolic hormone in the brain because of its anorexigenic properties (120). Insulin also strengthens the signals induced by leptin through JAK2 and SHT3 in the hypothalamus (121). Leptin and insulin resistance has recently been reported in the hypothalamus of diabetic mice, thereby opening new ways for a better understanding of insulin resistance and type 2 diabetes (33).

### Role in reproduction

Fertility is closely dependent on energy reserves, since negative alterations of energy homeostasis produce changes in the control of reproduction (122) through the hypothalamic–pituitary–gonadal axis (123). By contrast, an abundance of nutrients enables the reproductive process and survival of offspring. To control the relationship between the reproductive function and metabolic activities, several hormones such as insulin are involved. This hormone acts at several levels in the interplay between the hypothalamus, pituitary gland, and gonads. Thus, when hypothalamic pieces were perfused with low concentrations of insulin, a stimulatory effect on LHRH secretion was observed, which was dependent on the availability of glucose (124). However, high levels of glucose alone did not modify the release of LHRH (124). In the same way, the intracerebral infusion of insulin also increased the luteinizing hormone (LH) pulse frequency, but glucose infusion did not modify gonadotropin secretion (125). In diabetic animals, low levels of circulating insulin were accompanied by a reduced LH release (124), while the central or peripheral insulin administration restored LH pulse frequency (126). Other authors have found that low levels of circulating insulin in diabetic rats decrease both gonadotropin-releasing hormone (GnRH) release from the hypothalamus and the response of pituitary LH-releasing cells to GnRH (126). These results led Tanaka et al. (127) to propose that intracerebral insulin is a key regulator of pulsatile GnRH secretion in diabetic sheep. However, other authors have not ruled out the effects of glucose in the process, indicating that LH secretion is not wholly dependent on insulin activity because specialized glucodetectors in the hypothalamus can also modulate GnRH secretion, depending or not on insulin (128).

### Effect on cell proliferation and differentiation

Although the role of insulin as a neurotrophic agent in the adult brain is little known, the trophic function of insulin referred to proliferation, differentiation, and neurite growth has been reported in developing nervous systems. In fact, the systemic administration of insulin increases the ornithine decarboxylase activity in the brain of neonatal rats, which is an indicator of growth stimulation, indicating in this case that insulin was involved in the regulation of brain development (129). In addition, with the observation that the number of IRs increases during cell differentiation in the developing brain (130), IR signaling was suggested to play an important role in neuronal proliferation during development, which was confirmed when it was shown that IRS-2 mediated the effects of insulin on brain growth (131), as well as on the outgrowth, maturation, and regeneration of axons (132), as well as on neurite growth (18).

Nevertheless, the most abundant evidence on the neurotrophic effects of insulin has been obtained by *in vitro* studies using different neural cell cultures. Thus, it was determined that insulin-stimulated nucleotide incorporation in rat brain (133) and induced the growth and differentiation of a fraction of neurons isolated from the chick forebrain (134). The effects of insulin on growth and development mediated by IRs have been reported in both neurons and glial cells (135, 136), where both the number and the activity of the IRs may be regulated oppositely depending on cell type. On the other hand, insulin and IGF2 are necessary for NGF to stimulate neurite formation (137), while this effect is not observed in their absence. It has been also reported that these actions were dependent on the presence of astrocytes (138). In fact, astrocytes are known to modulate neuronal functions, and they may contribute to the cerebral actions of insulin, including cell growth. Thus, it has been reported that insulin induces the proliferation of both cultured rat (139) and human (140) astrocytes, in which the expression of several key proteins of insulin signaling was shown to increase.

In cultured fetal neurons, insulin increased both ribosomal protein S6 phosphorylation (136) and PKC-epsilon activity via a mechanism that does not involve the translocation of the enzyme from cytosol to the membrane (141), which could be closely related to neurite outgrowth (142, 143). This hormone also modulates the growth of neuronal cells by activating other protein kinases, such as phosphatidylinositol 3-kinase (PI3K) (144). Likewise, insulin increased the protein expression of the dendritic scaffolding protein post-synaptic density-95 (PSD-95) in hippocampal area CA1 through the activation of the PI3K/mTOR pathway, providing a molecular mechanism that could explain the effect of insulin on synaptogenesis and on the modulation of the synaptic function in area CA1 (145), as well as on the regulation of dendritic spine formation and excitatory synapse development in hippocampal neurons (146). Other proposed mechanisms for explaining the effect of insulin on neurite formation could be the upregulation of tau protein, the main microtubule-associated protein in the CNS that participates in the axon/neurite growth, which also involves the activation of the PI3K/mTOR pathway, or the upregulation or stabilization of tubulin mRNA followed by an increase in protein levels (147). It has also been reported that endogenous insulin synthesized by neurons was capable of promoting neurofilament distribution, which was abolished in the presence of IR inhibitors or antibodies against insulin (148).

On the other hand, both the proliferation and differentiation of multipotent neural stem cells are regulated by insulin, while the withdrawal of this hormone causes non-apoptotic autophagic cell death (130). Likewise, a reduction in the activation of the PI3K/Akt pathway has proven to be critical to the cell survival signaling of differentiated human neurons and human-derived neural stem cells (hNSC), which unlike the NSCs of rodent origin are extremely sensitive to insulin, and growth healthily in a narrow range of relatively low insulin concentrations (149). It is now accepted that IR pathways function as an integrating factor that correlate neuronal differentiation with nutritional information, to the extent that the degree of differentiation adapts to modifications of the nutritional state (150).

### Neuroprotective effects

Insulin is a potent neuroprotective agent that acts mainly against apoptosis, beta amyloid toxicity, oxidative stress, and ischemia. It has been reported that the antiapoptotic effect of insulin is dependent on the PI3K pathway, but not on the mitogen-activated protein kinase (MAPK) route, because the inhibition of mTOR activity by rapamycin avoids the antiapoptotic effects of insulin, suggesting that the protein p70SK, one of the downstream targets of the PI3K/Akt/mTOR pathway, may be one of the mechanisms through which insulin prevents apoptosis (151). Several authors have reported that insulin protects against beta amyloid-induced cell death. They have shown that the formation of Aβ fibrils is prevented by insulin, and suggest that Aβ damage may be caused through the regulation of Aβ fibrillation (152).

Insulin antagonizes the deleterious effects of oxidative stress in the CNS. Lipid and protein oxidation occur as a consequence of oxidative stress, which may alter proteins such as GLUT-3, modifying glucose uptake and then lactate accumulation, acidosis, and mitochondrial dysfunction (153). By stimulating glucose uptake and pyruvate formation, insulin restores intracellular ATP formation as well as reduces oxidative stress (154). Likewise, GABA and glutamate uptake are decreased under oxidative stress, which induces the accumulation of these neurotransmitters in the extrasynaptosomal space, while the addition of insulin reverses these changes (155). On the other hand, under situations of severe oxidative stress, the elevation of uric acid by insulin might provide some antioxidant benefit because uric acid, glutathione (GSH), and vitamins C and E are important as components of a neuronal antioxidant pool (156).

Two mechanisms have been proposed as being involved in insulin protection against ischemia; one by the direct effect of insulin on the brain tissue and the other one by an indirect mechanism in which insulin reduces peripheral glucose levels (157). As regards the former, it has been reported that insulin treatment increases the extracellular GABA during transient ischemia, independent of hypoglycemia, which can inhibit pyramidal neurons and protect them against ischemia (158). Another explanation is based on alterations of glucose metabolism and decreased lactic acidosis (159). Thus, insulin has a stimulatory effect on the Na^+^/K^+^ ATP pump, reducing both extracellular K^+^ and intracellular Na^+^, which may change the neuronal firing rate and its metabolic demands, while preventing water accumulation and the subsequent post-ischemic edema (160). On the other hand, as cerebral ischemia-reperfusion induces JNK1/2 phosphorylation, Bcl-2 expression, and caspase-3 cleavage in the rat hippocampus, and insulin reverses all the changes mentioned, it should be concluded that a cross-talk exists between Akt and JNK1/2 that could play a role in the anti-ischemic effects of insulin.

### Neuromodulatory effects

It is now widely recognized that insulin has neuromodulatory effects on mammalian CNS by acting both at electrophysiological levels, as well as on the concentration and function of certain neurotransmitters. Thus, it has been shown that insulin has direct and reversible electrophysiological effects on all of the recorded neurons *in vivo*, and these effects are highly dependent with respect to GABA pretreatment, being blocked by the co-administration of IR inhibitors (161). In addition, as the GABA receptor is a substrate of Akt phosphorylation, insulin may play an important role in the control of GABA receptor density in the post-synaptic domain (162). Insulin also affects intracellular ion concentrations by modulating the activity of certain ion channels. Thus, insulin in hypothalamic neurons activates K^+^ ATP channels producing membrane hyperpolarization, which has an inhibiting effect (163). In addition, insulin has stimulatory effects on Na^+^/K^+^ ATPase, producing an acute rise in intracellular Ca^2+^ concentration that triggers the release of neuropeptides (164).

Insulin may modulate neurotransmitter concentration through different mechanisms. Thus, insulin induces both the inhibition of norepinephrine and the stimulation of serotonin reuptake in neuronal cells (165, 166), which may increase glucose homeostasis through the interrelationship between brain IRs and the neurotransmitter function (130). Insulin also has modulatory effects on neurotransmitter receptor density. Thus, insulin reduced the increased number of dopamine receptors in striatal membranes from rats that were rendered diabetic with alloxan or streptozotocin (167), and in rats treated with haloperidol (168), but it had no effect on binding in normal rats. However, systemic insulin administration produced an increase in dopamine and serotonin levels in the CSF, while this hormone downregulated the α_2_-adrenergic receptors in hypothalamic neurons (169). On the other hand, insulin has a stimulatory effect on the uptake of amino acids by the neurons required for neurotransmitter synthesis (170).

### Insulin effects on cognition and memory

It has been widely reported that the peripheral or central administration of insulin by icv or intrahippocampal routes to experimental animals has positive effects on memory and learning processes (171). The improvement in these activities is related with an increase in both the IR expression and its signal transduction pathways in the hippocampus (172), and the loss of memory due to ischemic lesions in this structure can be avoided by insulin administration (173). Streptozotocin has been used to develop experimental models of type 1 diabetes. However, when injected directly at low doses into the brain, it produced a central resistance to insulin by interfering with the binding of the hormone to its own receptor and blocking insulin actions, which was related to deficits in memory and altered behavior (174). Besides, epidemiological studies have shown that both type 1 and type 2 diabetic patients have cognitive impairment and an increased risk of AD, mainly in older patients, and also that insulin administered to AD patients to keep glycemic levels constant can improve memory formation (175). Likewise, the systemic administration to healthy humans of insulin under euglycemic hyperinsulinemic conditions yields a significant improvement in verbal memory and selective attention.

It is believed that an event is fixed in the memory by modifications of the neuron networks based on the processes called long-term potentiation (LTP) and long-term depression (LTD) (176). The LTP process occurs when the presynaptic neuron excites the post-synaptic ones in a repetitive and prolonged manner, and then the depolarization of the post-synaptic neuron is reinforced and maintained for a long time. Accordingly, there is a significant increase in Ca^2+^ input, and the metabolic activities for this cation are prolonged, and as a result the memory is consolidated. The LTD is a compensatory process that in the post-synaptic neuron, still under the effect of a LTP, facilitates a decrease in transmission efficiency, permitting cell activity to return to the previous excitatory level, and then get ready to store new information. Memory and learning functions need LTP and LTD processes, but also the remodeling of the dendritic spine morphology and modifications in the cytoskeleton produced during synaptic transmission. These processes, involve the glutamate, as well as two of its receptors, AMPA and NMDA, in which neurotransmission is regulated by changing the amount of receptors present in the membrane, or by covalent modification of their subunit components. Thus, whereas LTP increases the post-synaptic density of the AMPA receptors, LTD is associated to a decrease. Likewise, the phosphorylation of these receptors increases the efficiency of the ionic channel during LTP, while dephosphorylation during LTD decreases it (177).

Insulin modulates glutamatergic neurotransmission at the synapses. This hormone induces the LTD process by decreasing the amount of AMPA receptors in the post-synaptic membrane. Besides, this process depends on the phosphorylation of the hormone receptor, PI3-kinase activation, and on a process of protein synthesis (178). Other authors reported that insulin also induces the phosphorylation of the GluR2 subunit in the AMPA receptors of hippocampal neurons, producing endocytosis and a decrease in the post-synaptic excitatory ability (179). There is also experimental evidence that insulin affects learning and memory through GABA receptors by stimulating the translocation of these receptors to the plasma membrane. This effect is abolished by the action of a PI3K inhibitor. Insulin also increases the functional GABA receptor expression on the post-synaptic and dendritic membranes of the CNS neurons (18). Likewise, NO has also been reported to be involved in insulin-induced memory improvement, since the administration of the NOS inhibitor L-NAME avoids insulin-induced memory improvement (180).

IGF1 increases the synaptic transmission in the rat hippocampus through a mechanism in which AMPA receptors and PI3K activity are involved (181). Furthermore, GH increases the expression of the subunits of the NMDA in the hippocampus, thereby facilitating LTP induction and improving the memory (182).

## Inflammation, Insulin Resistance, and the Brain

There is experimental evidence to indicate that inflammatory responses are closely associated with the development of insulin resistance in peripheral and central tissues, as well as to show that these processes are present in obesity and in type 2 diabetes, which may increase the risk or incidence of AD (183). Thus, high concentrations of interleukin IL-6 have been determined in the CSF of patients with AD (184), while studies in animals suggest that inflammation interacts with the processing and deposit of β-amyloid peptide (Aβ) (185), with low amounts of insulin producing anti-inflammatory effects (186). The administration of lipopolysaccharide increases in plasma concentrations of the C-reactive protein and pro-inflammatory cytokines IL-1β, IL-6, and TNF (187). TNFα and IL-6 also induce the activation on NFkβ and subsequent transcription of the pro-inflammatory genes TNFα, IL-6, and IL-1b (188). Increased levels of inflammatory cytokines alter hippocampal synaptic plasticity and the components of spatial learning (189). Furthermore, obesity induces a peripheral insulin resistance that is related with a marked elevation of pro-inflammatory cytokines and of free fatty acids. Chronic inflammation may contribute to the appearance of insulin resistance and type 2 diabetes, as well as to the association of AD and type 2 diabetes mellitus (T2DM) (190). In addition, the increased concentrations of TNFα and Aβ within the brain of obese hyperinsulinemic persons facilitates the formation of amyloid plaques (191). Obese and AD patients have CSF insulin concentrations lower than in control subjects, suggesting a reduction in both insulin transport across the BBB and hormone sensitivity (192).

TNF-α has both neurotoxic and neuroprotective effects, depending on the receptor subtypes TNF-R1 and TNF-R2. Thus, TNF-R1 is involved in pro-apoptotic actions, while TNF-R2 promotes cell survival. High levels of TNF-R1 and decreased concentrations of TNF-R2 have been described in the brain of AD patients (193). Altered levels of TNF-R1 and TNF-R2 have been reported in persons with diabetes and impaired glucose tolerance (194), which was normalized after a 3-week low calorie diet (195). The accumulation of glycation end products, oxidative stress, and the resulting brain cellular and molecular damage may contribute to diabetes-induced brain aging (196).

Alterations of some of the insulin signaling pathways such as PI3K/Akt and GSK-3 are recorded in central inflammation and insulin resistance (197). It is known that the PI3K pathway has a negative effect on IL-12 formation by dendritic cells, while GSK-3 is a tau kinase involved in hyperphosphorylation and in the modulation of Aβ metabolism (198). There are some connections between the insulin signaling pathway and this protein, as IR activation phosphorylates and inhibits GSK-3β (199), while in AD, GSK-3β activity is increased as well as in T2DM, and this process enables it to phosphorylate the IR and IRS-1, which decrease the phosphorylation of tyrosine residues of the IR and IRS-1 (200). In addition, the activation of STAT-3 in astrocytes and microglia depends on GSK-3β activity (201), while the inhibition of GSK-3 stimulates the production of anti-inflammatory cytokines such as IL-10, and decreases pro-inflammatory cytokines such as IL-1β, IL-6, and IFN-γ in response to toil receptors (202). These findings suggest that PI3K/Akt/GSK-3 play an important role in controlling inflammation, and that the inhibitory effect of insulin on GSK-3 activity shows how insulin controls inflammatory responses.

It is well accepted that neuroinflammation occurs in AD, with infiltrates of T-lymphocytes and monocytes that are responsible, together with microglia cells, for the increase in a number cytokines in the CNS (203, 204). Inflammation is needed for the expression of insulin resistance, as demonstrated by the inhibition of inflammatory pathways, which avoid diet-induced insulin resistance in experimental animals (205). In fact, the initial part of high-fat diet-induced insulin resistance is independent of inflammation (206), while the chronic states are dependent on inflammatory processes (207). Accordingly, TNF-α reduces the tyrosine-kinase of the IR, resulting in insulin resistance, and the reduction in other mediators such as SOCS-3 inhibits IRS-1 (208). In addition, the activation of JNK by cytokines, inflammatory mediators, and fatty acids phosphorylate IRS-1, modifying insulin signaling. Thus, animals lacking JNK are protected against the high-fat diet (209). On the other hand, JNK inhibitors improve the neuroinflammatory response, while JNK activation promotes neuroinflammation and facilitates insulin resistance (210). Sartorius et al. (211) suggest that the activation of neuroinflammatory reactions may be responsible for the high-fat diet-induced insulin resistance in the brain, since the prevention of a neuroinflammatory response blocks this resistance (211, 212). In the same way, the icv administration of TNF-α produces hypothalamic inflammation, as well as the expression of the phenotype present in type 2 diabetic patients with high insulin levels and altered insulin signaling in peripheral tissues. It is accepted that central insulin resistance can favor an adaptive increase in food intake that facilitates the peripheral alteration of glucose homeostasis. Alternatively, the elevation of free fatty acids is a signal to increase the release of pro-inflammatory cytokines, which then activates the inflammatory signaling pathways responsible for insulin inhibition signaling and the promotion of insulin resistance (213).

Impaired insulin signal transduction with reduced tyrosine-kinase activity of the IR has been reported in the brain cells of AD patients. Moreover, the expression of insulin and IGF1 mRNA and protein levels, their own receptors, and the downstream signaling elements are decreased in the brain of AD patients (214). Insulin resistance is thus associated with reduced responses to insulin signaling in the IR/IRS-1/PI3K and greatly with IGF1 in the IGF1R/IRS-2/PI3K signaling pathways. Reduced insulin responses peaked at the level of IRS-1, and were consistently associated with basal elevations of IRS-1 phosphorylated at serine 616 (IRS-1 pS^616^) and IRS-1 pS^636/639^. These potential biomarkers of insulin resistance increased significantly from normal cases, through mild cognitively impaired cases, to frank AD cases, regardless of APOE-4 status (104). Related with this insulin insensitivity, the protective role of this hormone against Aβ accumulation is reduced, at the same time as the expression and function of insulin are downregulated by Aβ deposits (215). Thus, Aβ peptides inhibit the binding of insulin to its receptors (216), reduce receptor autophosphorylation, and impair insulin-induced signaling pathways (217). The alterations of these effects in AD and T2DM patients interfere with the neuroprotective actions of insulin, facilitating the brain’s susceptibility to neurodegeneration (218). Both brain insulin and IGF1 resistance are considered an early and common feature of AD, which seem to be closely associated with the IRS-I dysfunction triggered by Aβ oligomers that promote cognitive decline (104).

## Relationship between Diabetes and Alzheimer’s Disease

**Figure 3:**
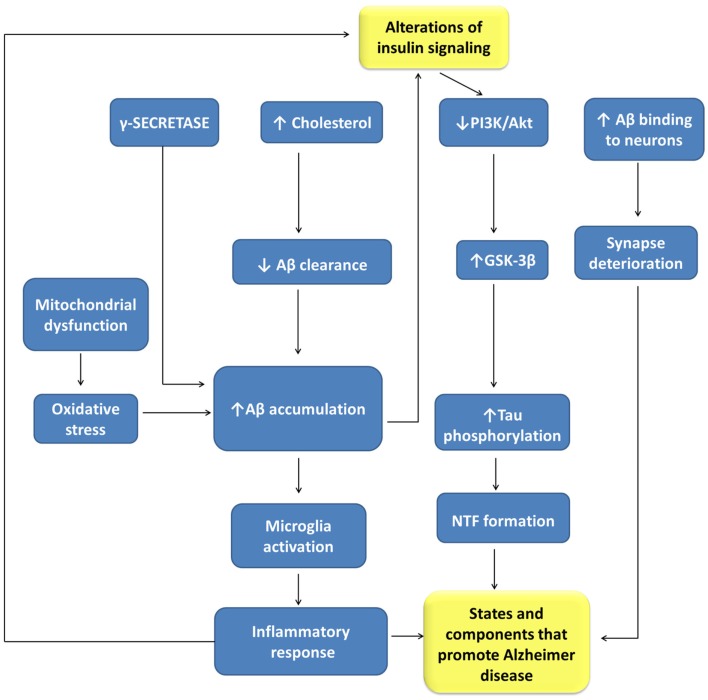
**Relationships between alterations of insulin signaling and Alzheimer disease pathogenesis**. Aβ, beta amyloid-peptide; GLUT-3, glucose transporter isoform-3; GSK-3β, glycogen synthase kinase 3-β; NFT, neurofibrillary tangles; PI3K, phosphatidyl inositol 3-kinases.

There is sound evidence about the role of insulin in brain functions, as well as of the close relationships between AD and T2DM (219), two highly prevalent nosological entities (Figure 3). AD is a neurological disorder that causes profound memory loss and progressive dementia, with histological manifestations of amyloid plaques, neurofibrillary tangles, and amyloid angiopathy, accompanied by widespread loss of neurons and synapses (220). There are more than 30 million people suffering from AD, with this number increasing very quickly and expected to exceed 120 million by 2040 (197). T2DM is characterized by impaired insulin secretion and by a resistance to the action of this hormone. It has been estimated that there were 250 million diabetic patients worldwide in 2010, with 90% of the patients having T2DM (221). Aging is a high-risk factor for both AD and T2DM. Accordingly, T2DM is a disease that seriously affects the quality of life and longevity of elderly people, although in recent years it also affects obese young people.

Both disturbances share many pathophysiological features, such as insulin resistance, amyloid aggregation, inflammatory stress, and cognitive disturbances that suggest common or related pathogenic processes. Insulin resistance is a risk factor for AD, being a common feature of AD patients with or without T2DM. Besides peripheral insulin resistance, this alteration may be present in the brain accompanied by IGF1 resistance, and IRS-1 and IRS-2 dysfunction, potentially triggered by Aβ oligomers and cognitive decline.

Interestingly, peripheral insulin resistance begins a long period of time before the appearance of a frank T2DM, which might permit a preventive therapeutic treatment during this period (222). To know whether peripheral or central insulin resistance are components of the same disease or are expressed independently of one another is a matter of potential interest. What’s more, the existence of AD in patients without T2DM could be related to a lack of time to develop the diabetic process, or be the consequence of different nosological entities. AD is a progressive neurodegenerative disease, and when the symptoms and signs of cognitive dysfunction appear, the disease has already been present for many years. Accordingly, four stages for AD (223) have been proposed: a pre-disease stage without detectable pathophysiological alterations, a preclinical stage with pathophysiological alterations but without cognitive alterations, a stage of pre-dementia or mild cognitive impairment (224), and the dementia stage. It would be of great interest to know whether these stages coincide in time with the predicted stage in the peripheral tissues during the long period of insulin resistance. This period of time during prediabetes or the four stages of AD may constitute an opening for the development of preventive programs or new therapeutic approaches.

Many authors have suggested that insulin alterations and changes in glucose metabolism may condition the risk of developing dementia (225, 226). A recent study indicates that the altered expression of genes related to T2DM and AD brains (227) is a result of AD pathology, which may be exacerbated by insulin resistance or DM (227). This statement can be stressed because cognitive deficits are associated with insulin signaling abnormalities. The close relationship between these two pathological disturbances, because of the common presence of insulin resistance, has led to the use of the term type 3 diabetes, which means it is considered a neuropathogenic expression of AD. However, this entity cannot be included (228) within the classic concept of diabetes, because AD patients are not hyperglycemic, as happens with both T1DM and T2DM, nor does insulin stimulate glucose uptake in the brain, by contrast with the strong stimulatory effect observed in muscle, fat, and the liver. Nevertheless, an insulin-resistant brain state (229) exists as a variant of the hormone manifestations in several peripheral tissues, with different properties as corresponds to the brain functions. Insulin resistance in the peripheral tissues could facilitate insulin resistance in the brain by reducing brain insulin uptake and by increasing levels of Aβ (230). It seems that insulin resistance is a prior and common manifestation of these diseases, which over a long period of time deteriorate central or peripheral tissues before the appearance of diabetes and AD. This period, which lasts around 10–15 years, and is referred to as prediabetes, is more frequent in older people at a time when the incidence of AD is increasing significantly. The prevalence of prediabetes and diabetes in AD is of 81% in the USA (226), in which peripheral insulin resistance was accompanied with or without T2DM. The long duration of prediabetes might explain why brain insulin resistance in some cases of AD may be found without diabetes and/or the high prevalence of both prediabetes and AD in the elderly.

Both experimental and epidemiological studies provide evidence to show that the risk of AD dementia or vascular dementia is increased in diabetic patients (231). Thus, large epidemiological series have reported the association between insulin resistance or DM and the risk of AD, albeit independent of the APOE-4 phenotype (232). Furthermore, hyperinsulinemia and hyperglycemia caused by insulin resistance accelerate the formation of neurite plaques (233). The results from a nationwide case-control study in Finland (234) show that individuals with AD are more likely to have a history of medically treated diabetes than the older population in general, and that the association is independent of vascular diseases.

Most studies have focused on the role of type 2 diabetes, while the association between type 1 diabetes and alterations of CNS has received less attention (235). Then, T2DM is clearly associated with AD, while T1DM is more related to other brain alterations (235–240). However, learning and memory impairments and deficits in mental flexibility and problem-solving are more frequent in patients with type 1 diabetes than in the general population (241, 242). Interestingly, these alterations improve after the establishment of insulin therapy, when the glycemia is controlled (243, 244). These alterations may have a morphological explanation, since patients with type 1 diabetes have a reduced number of dendrites in the gray matter (245). Accordingly, intranasal insulin administration in diabetic mice reverses morphological and cognitive alterations (246). In addition, type 1 diabetes and type 2 diabetes are associated with brain atrophy and cognitive impairments, which are prevented by insulin and IGF1 (247).

Besides the studies cited above establishing a risk of AD development in diabetic patients, there are other reports with opposite findings in which they have stated that although diabetes produces alterations in cognitive performance or increases the risk of dementia, there is no association between DM and AD (248, 249).

In AD patients, the CSF concentrations of insulin are decreased, while the blood plasma concentrations are raised, which is more evident in advanced stages of AD and in patients without APOE-4 allele (197). High levels of circulating insulin may be the consequence of hormone resistance, while the reduction in CSF insulin may be related to a decrease in insulin clearance and/or to a reduction in insulin uptake from a peripheral source through the BBB.

Several experimental evidences support the link between AD and DM through mitochondrial alterations and oxidative stress, altered energy and glucose metabolism, cholesterol modifications, and dysfunctional protein O-GlcNAcylation (197). Since insulin avoids the reduction in mitochondrial oxidative phosphorylation and any increase in oxidative stress that protects against Aβ-protein toxicity, we can infer that diabetes is a risk factor for AD development. In fact, diabetes induces alterations in the mitochondrial antioxidant defense, facilitating brain susceptibility to Aβ toxicity (250).

Impairments in energy and glucose metabolism, manifested as reduced cerebral glucose and oxygen utilization, are present both in non-diabetic and diabetic patients with AD, indicating another link between these two nosological entities (223). PET technology has been used to find a depressed glucose metabolism in the temporal–parietal cortex, posterior cingulate cortex, and the frontal areas of AD patients. The administration of glucose to these patients therefore improves memory alterations (251, 252), although this effect seems to be due more to insulin than to hexose. At this point, it is important to remember that glucose metabolism dysfunction increases the risk of cognitive disturbances in the elderly.

Contributions to cerebral glucose hypometabolism may be related to glucose transportation abnormalities, intracellular glucose metabolic disturbances, and the altered functional status of thiamine metabolism. AD patients have decreased GLUT-1 and GLUT-3 expressions (253), especially in the cerebral cortex. The dentate gyrus of the hippocampus also has reduced GLUT-3 expression. Liu et al. (254) have explained that glucose hypometabolism results in both reduced glucose transporter expression and the decreased O-GlcNAcylation of tau. They proposed that hypometabolism in the brain reduces the O-GlcNAcylation of tau that conversely increases its phosphorylation, which induces the NFTs that underlie the cognitive deficits of AD subjects. Three key enzymes in the Krebs cycle and pentose phosphate pathway (PPP)-pyruvate dehydrogenase complex, α-ketoglutarate dehydrogenase complex, and transketolase, and their common coenzyme thiamine diphosphate (255), recorded altered levels in the brain of AD patients. These findings suggest the relevant roles of mitochondrial dysfunction and impaired thiamine-dependent processes in the cerebral glucose hypometabolism of AD.

Impaired cerebral glucose metabolism is a pathophysiological feature that may even precede pathological alterations by decades (222). Accordingly, Chen and Zhong proposed the hypothesis that impaired cerebral glucose metabolism, mainly thiamine metabolism and insulin resistance, could promote Aβ accumulation and tau hyperphosphorylation, as well as many other pathogenic factors that might contribute to the pathological dysfunction of the brain in AD. These pathophysiological cascades include inflammatory factors, mitochondrial dysfunction, and oxidative stress, Advances Glycation End products (AGEs), apoptosis, excitotoxicity, and the hyper-activation of protein kinases. All these factors are involved in cognitive dysfunction (223).

In addition, changes in brain cholesterol metabolism facilitate the interactions between DM and AD pathogenesis. Thus, cholesterol accumulation alters beta cell functions and insulin secretion (256), as well as insulin-stimulated cholesterol biosynthesis, and controls its circulating levels (257), which form a strong bond between hypercholesterolemia and T2DM. In addition, elevated levels of circulating cholesterol increase the risk of AD (88) because it modulates Aβ synthesis, inhibits the clearance of Aβ, and potentiates the interaction of Aβ with neuronal membranes (10). Furthermore, Aβ binds to cholesterol to catalyze the formation of oxysterols, products with highly neurotoxic properties, which alter the insulin signaling pathway by inhibiting the phosphorylation of the ERK/Akt route (258, 259). It has been reported that hyperglycemia and hyperinsulinemia activate the O-GlcNAcylation of proteins included in the insulin signaling pathway, facilitating insulin resistance (260, 261). Studies on autopsied frontal cortices have found that the protein content and activity of the brain insulin/PI3K/Akt signaling pathway are decreased in T2DM and AD patients, and more so in AD-T2DM patients (262). The reduced brain insulin/PI3K/Akt pathway leads to the overactivation of GSK-3β calpain-1 and downregulation of O-GlcNAcylation, which promoted altered tau hyperphosphorylation and neurodegeneration (262). These data suggest that the alteration of GlcNAcylation may be another mechanism by which the predisposition for AD is increased by DM.

Other situations, such as the formation of amyloid plaques, altered Aβ metabolism, and tau hyperphosphorylation, are favored by the interactions of AD and DM. Aβ are the components of the amyloid deposits in the AD brain, while the protein deposited in the islets of Langerhans is the islet amyloid polypeptide IAPP. Both proteins are able to form amyloid aggregates (263), with the Aβ protein being toxic for neurons, and IAPP toxic for pancreatic islet cells (224). These two proteins have a high sequence similarity, where the chaperone protein pathway preventing IAPP and Aβ aggregation may be common and act on both of them. It has been suggested that the decreased capacity of this shared chaperone protein is responsible for the development of AD and T2DM (226). This means that islet amylogenesis is increased in patients with AD, and that the density of neurite plaques and their diffusion are positively related to the duration of diabetes (226).

Aβ is degraded and cleared by several proteases that avoid the accumulation of Aβ. IDE is a metalloendopeptidase that inactivates peptides such as insulin, IGFs, glucagon, and others. The alterations in insulin signaling also affect the Aβ metabolism, with Aβ and phosphorylated tau accumulation associated to neuronal loss and neurite degeneration. These effects are more significant in T2DM (264). Some data show that the activation of insulin signaling in CNS can upregulate IDE activity, and may correct the IDE defects present in AD (265, 266). Insulin also stimulates the internalization of Aβ oligomers and inhibits their binding to neurons, and thereby protects synapses against Aβ oligomers.

Neurofibrillary tangles are mainly composed of hyperphosphorylated tau molecules (267), which may be cleavaged by several proteases such as caspases and calpains, which play an important role in AD pathology (268). Alterations of insulin signaling in types 1 or 2 diabetes increase tau phosphorylation and tau cleavage, promoting AD pathology (235). Insulin resistance in T2DM, and the corresponding peripheral hyperinsulinemia, reduces insulin transport through the BBB, and the systemic insulin deficiency in T1DM increases tau phosphorylation (235).

Besides in AD, there are several mental diseases such as Huntington’s disease, depression, and schizophrenia in which insulin disturbances play a pathogenic role (197).

We should propose that resistance to the central action of insulin may be a meeting point between T2DM and AD and the slow pathological progression of both nosological entities should facilitates the deterioration of neurons mainly in the hippocampus and the cerebral cortex. Further studies are needed to accept or refuse this hypothesis, as well as with the possibility that AD may be caused though a slow deteriorating effect by microbial infection (269) by some viruses and bacteria; recently has been also proposed that fungal infections may represent a risk factor or possibly the cause of AD (270, 271). All the entities cited above have in common a slow deteriorating effect on CNS and represent an interesting matter to be further investigated. It should be also interesting to know whether patients with AD and cerebral infection with some viruses, bacteria, or fungus have central resistance to the action of insulin.

## Therapeutic Approaches

Scientific advances in cognitive functions, brain metabolic, and energy control, have provided new openings for trials on insulin resistance and on therapeutic approaches associated to nosological entities such as AD and DM. Therapeutics for AD treatment based on amyloid hypothesis (272) and tau hyperphosphorylation hypothesis (273) have been developed, but they perform poorly. Likewise, antioxidants, anti-inflammatory, and neuroprotective agents have been used with negative results (274, 275).

As thiamine deficiency is frequent in AD patients, drugs targeting altered thiamine metabolism have been used with conflicting results. Based on the hypothesis of multiple pathogenic cascades induced by glucose metabolism dysfunction, “cocktail therapies,” or drugs acting at multiple pathogenic cascades have been developed for AD (276).

Advances in the knowledge of preclinical AD and T2DM have provided a major stimulus for the development of treatments for preventing the pathogenic events of these disorders, focusing mainly on reducing brain insulin resistance.

At present, the main therapeutics available are insulin-sensitizing agents, metformin and the peroxisome proliferator-activated receptor gamma (PPARγ) agonists, and the incretin insulin mimetics molecules, glucagon-like peptide-1 (GLP-1), and gastric inhibitory peptide (GIP), which are insulin secretagogues. In addition, the beneficial effects of intranasal insulin or GLP-1 administration to patients with mild cognitive impairment (224) or T2DM have to be considered.

The biguanide metformin is one of the more used agents in T2DM that improves fasting insulin levels and the control of insulin on hepatic glucose production. Considering that its major action is on the liver, it has been reported in recent years that it can also cross the BBB (277), which given its insulin-sensitizing properties suggests that it may play a role in insulin resistance and dementia. In fact, an epidemiological study reported that treatment with metformin reduces the incidence of dementia in diabetic patients (278).

In addition, PPAR gamma agonists have been used in the treatment of T2DM because they improve insulin sensitivity (279), increasing the function of adipose tissue, and moving triglycerides and fatty acids away from the liver and muscle. Furthermore, they reduce both Aβ accumulation and neuroinflammation (280, 281), which may improve pathologies related with AD and T2DM, such as MCI (Mild Cognitive Impairment) associated with insulin resistance. Treatment with the PPAR gamma agonist rosiglitazone improved attention and memory, reducing fasting insulin levels in patients in the first stages of AD (282). However, the increased incidence of heart failure in patients treated with PPARγ agonists has reduced their use in diabetic patients, which has forced the development of new procedures and drugs.

Glucagon-like peptide-1 is an incretin that works as an insulin secretagogue in a glucose-dependent manner. Gliptins that delay the degradation of GLP-1 or exenatide and liraglutide or GLP-1 mimetics, with a more stable structure, reduce the degradation of GLP-1, and are therefore very useful in diabetes therapy. As metformin, GLP-1 mimetics readily cross the BBB and they induce several actions through GLP-1 brain receptors. Both exenatide and liraglutide were found to antagonize processes related to neurodegeneration and AD progression in mouse models (283). This may be because these agents are neuroprotectants (284, 285) that decrease oligomeric Aβ, neuritic plaque load, and microglial activation (283, 286). Furthermore, they stimulate neurogenesis and improve object recognition and spatial memory (283, 287). These GLP-1 mimetics reduce insulin resistance in MCI and AD, which suggests they could be used in the treatment of dementia with or without diabetes.

Intranasal insulin administration has a beneficial effect on patients with MCI or T2DM. Excellent scientific contributions have been done in human patients and others in animal models (240, 288–290). This procedure has the advantage of avoiding certain shortcomings in insulin transport through the BBB, added to the fact it does not produce hypoglycemia, as sometimes happens after peripheral insulin administration. This kind of treatment should be of interest for attenuating central insulin resistance, but it was abandoned because of secondary complications. In addition, GLP-1 analogs through intranasal administration could be potential therapeutics for AD (291).

Certain molecules involved in the transduction of signals induced by insulin might be targets for new therapeutic approaches in the future. The desensitization of insulin signaling by IRS-1 Ser/Tht phosphorylation should play an important role in insulin resistance. As only phospho-Ser/Thr on IRS-1 can have negative or positive effects on the insulin signaling of healthy and diseased tissues, those molecules might play an important pathophysiological role (292). Whether or not IRSs constitute a drug target for the treatment of insulin resistance and gaining a comprehensive understanding of Ser/Thr phosphorylation will help to explain the pathophysiological processes that facilitate insulin resistance.

## Conflict of Interest Statement

The authors declare that the research was conducted in the absence of any commercial or financial relationships that could be construed as a potential conflict of interest.
